# Acute hepatitis B infection escaping vaccination: A case report

**DOI:** 10.1097/MD.0000000000048735

**Published:** 2026-05-29

**Authors:** Sonja E. Leonhard, Daphne N. Fioole, Kimberley S.M. Benschop, Roel P.J. Willems, Mariska Petrignani, Bettie C.G. Voordouw, Leonard C. Smeets, Hans L. Zaaijer

**Affiliations:** a Department of Medical Microbiology, Reinier Haga MDC, Delft, The Netherlands; b Department of Medical Microbiology and Infectious Diseases, Erasmus MC, Rotterdam, The Netherlands; c Department of Infectious Disease Control, GGD Haaglanden, The Hague, The Netherlands; d Department for Infectious Disease Research, Diagnostics and Laboratory Surveillance (IDS), Centre for Infectious Disease Control (CIb), National Institute for Public Health and the Environment (RIVM), Bilthoven, The Netherlandss; e Department of Medical Microbiology and Infection Control, Amsterdam UMC, Amsterdam, The Netherlands; f Department of Blood-Borne Infections, Donor Medicine Research, Sanquin Blood Supply Foundation, Amsterdam, The Netherlands.

**Keywords:** case report, hepatitis B virus, mutation, vaccine escape

## Abstract

**Rationale:**

Hepatitis B virus (HBV) is an important cause of acute and chronic infectious hepatitis globally. Vaccines against HBV are widely available and generally lead to lifelong protection against chronic infection, disease, and infectivity. Vaccine failure, in which a person fails to form sufficiently high antibody titers, occurs in 5% to 10% of the population. Vaccine failure due to mutations in the virus, enabling vaccine escape, on the other hand, is very rare.

**Patient concerns:**

A 72-year-old man presented with heartburn complaints. In clinical examination, an enlarged liver was found, and subsequent testing showed abnormal liver function. He was fully vaccinated against HBV.

**Diagnoses:**

Positive test results for hepatitis B surface antigen, immunoglobulin G and immunoglobulin M antibodies against hepatitis B core antigen, and antibodies against hepatitis B e and surface antigen were found. HBV DNA was positive (14,000 IU/mL). These results indicated an acute HBV infection.

**Interventions:**

A rare mutation in the S-gene of HBV, D144A, was found.

**Outcomes:**

The patient recovered without treatment.

**Lessons:**

We describe a case of acute HBV infection in a fully vaccinated person with high post-vaccination titers due to a rare mutation in the S-gene of HBV, which has previously been associated with vaccine escape. We describe the epidemiology of such mutations and the public health consequences of discovering such a strain in the community.

## 1. Introduction

Worldwide, approximately 250 million people are living with hepatitis B virus (HBV) infection, and approximately 1.3 million people die each year due to complications, including liver failure, cirrhosis, and hepatocellular carcinoma.^[1,2]^ In the Netherlands, the overall prevalence of chronic HBV infection in adults is estimated at 0.34%, corresponding to approximately 50,000 infected individuals.^[3]^ Vaccination is the most effective prevention. Safe and effective HBV vaccines have been available since 1982. A national immunization program targeting high-risk groups was successfully implemented in the Netherlands in 2003, and HBV vaccination has been incorporated in the National Child Immunization Program since 2011. The internationally accepted threshold for long-term protection against HBV infection is a level of antibodies against hepatitis B surfaceantigen (anti-HBs) of at least 10 IU/L at 1 to 3 months post-vaccination.^[4]^ Vaccine failure, other than non- or low response to vaccination, has rarely been described, and can be attributed to either failure of the (long-term) immune system (such as waning immunity) of the vaccinated person, or mutations in the virus enabling vaccine escape.^[5]^ Here, we describe a rare case of acute HBV infection in a fully vaccinated person, with documented presence of >1000 IU/L anti-HBs antibodies 6 weeks after vaccination, 21 years before the current HBV infection.

## 2. Case report

A 72-year-old retired policeman without a relevant medical history was seen by his general practitioner because of heartburn complaints. During physical examination, an enlarged liver was found. Liver function tests were abnormal, with an elevated gamma-glutamyltransferase and bilirubin and a strongly elevated aspartate aminotransferase and alanine aminotransferase. Serology for HBV, hepatitis A, hepatitis C, hepatitis E, cytomegalovirus, Epstein–Barr virus, and human immunodeficiency virus was subsequently done using the Alinity (Abbott) I-6 module (see Table 1).

**Table 1:** Laboratory results.

Test	Date						Unit	Reference values
	12/08/24	13/08/24	16/08/24	30/08/24	13/11/24	31/12/24		
Liver function tests								
ASAT	2483		690	66	43		U/L	0–35
ALAT	2966		1906	138	28		U/L	0–45
AF			196	107	70		U/L	0–120
Gamma-GT	140		124	57	17		U/L	0–55
Bilirubin conjugated	76		52	15			µmol/L	<5
Bilirubin total	90		62	22	13		µmol/L	<21
Albumin			36	38			g/L	35–50
Infection serology								
HBsAg	946.8	684.7	3.3			0.2	Ratio	<0.9
Anti-HBs titer		345	342			33	IU/L	<10
Anti-HBc	3.1	3.3	3.4			5.9	Ratio	<0.9
HBc IgM		37.1					Ratio	<1.0
HBeAg		Neg	Neg			Neg	Ratio	
Anti-HBe		Pos	Pos			Pos	Ratio	
HDV IgG	Neg							
CMV IgG		203					U/mL	<1.0
EBNA abs		>600					U/mL	<5
HAV IgM		Neg						
HAV abs		Pos						
HCV abs		Neg					Ratio	<0.9
HEV IgM		Neg						
HEV IgG		Neg						
HIV abs			0.1				Ratio	<1.0
HIV p24			0.2				Ratio	<1.0
HBV DNA		1.4 × 10^4^	52				IU/mL	

Serology was done using the Alinity (Abbott) I-6 module.

abs = antibodies, AF = alkaline phosphatase, ALAT = alanine transaminase, anti-HBc = antibodies against hepatitis B core antigen, anti-HBe = antibodies against hepatitis B envelope antigen, anti-HBs = antibodies against hepatitis B surface antigen, ASAT = aspartate aminotransferase, CMV = cytomegalovirus, EBNA = EBV nuclear antigen, EBV = Epstein–Barr virus, Gamma-GT = gamma-glutamyltransferase, HAV = hepatitis A, HBc IgM = IgM antibodies against hepatitis B core antigen, HBsAg = hepatitis B surface antigen, HBV = hepatitis B virus, HCV = hepatitis C, HDV = hepatitis delta virus, HEV = hepatitis E, HIV = human immunodeficiency virus.

Positive test results for hepatitis B surface antigen (HBsAg), immunoglobulin G and immunoglobulin M (IgM) antibodies against hepatitis B core antigen (HBc IgM) and antibodies against hepatitis B e antigen were found. Notably, anti-HBs antibodies were detectable too (345 IU/L). HBV DNA was tested with realtime-polymerase chain reaction and found positive (14,000 IU/mL HBV DNA). These results indicated an acute HBV infection.

**Figure 1.**
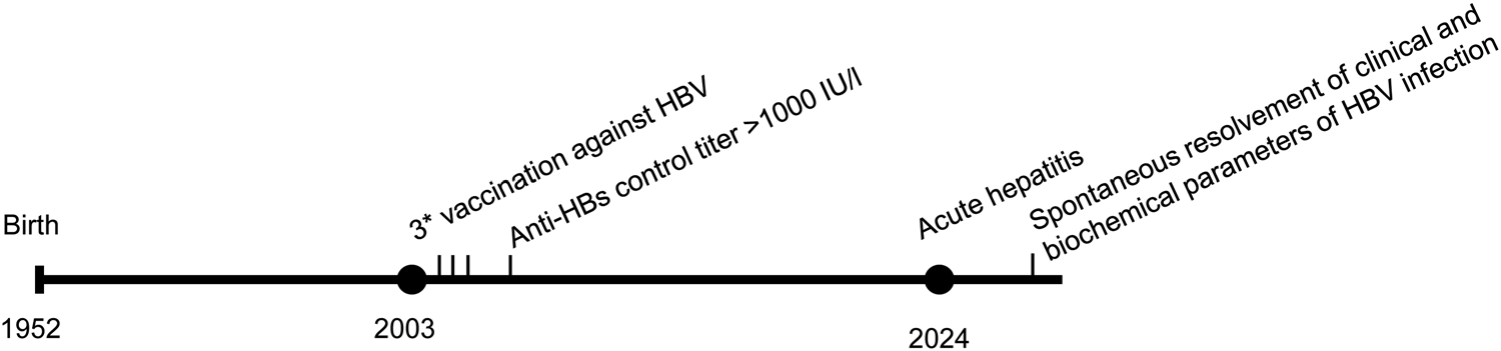
Timeline of vaccination and infection. Anti-HBs = antibodies against hepatitis B surface antigen, HBV = hepatitis B virus.

However, this patient had been fully vaccinated against HBV in 2003, with 3 dosages of Engerix-B and an anti-HBs titer of >1000 IU/L (Fig. 1), as documented by the municipal health service and his vaccination certificate. When asked, the patient indicated that he was vaccinated because of his work. The patient is married to a woman and denied recent risk behavior for contracting HBV infection, including sexual contacts with either men or women, blood transfusions, tattoos, or other potential blood-spilling contacts.

Blood samples were sent to the National Institute for Public Health and the Environment for genotyping of the near-complete genome using Minion sequencing.^[6]^ The strain was characterized as genotype A2, which is endemic in the Netherlands, mostly among men who have sex with men (MSM).^[7]^ In this gene, a D->A mutation was found at amino acid position 144.

The patient was referred to the gastroenterologist by his general practitioner for follow-up of his acute HBV infection. HBsAg and HBV DNA decreased spontaneously within a few days, and at 3 months follow-up, his liver test abnormalities and HBsAg had completely normalized (Table 1). His clinical condition also improved spontaneously.

### 2.1. Surveillance of mutations associated with vaccine escape

**Figure 2.**
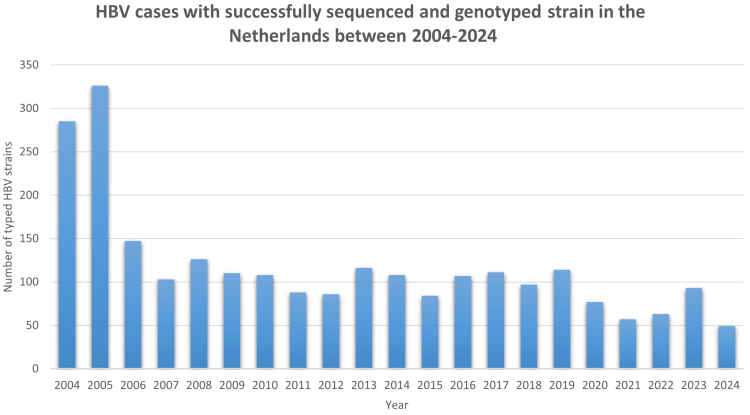
HBV cases with successfully sequenced and genotyped HBV strain in the Netherlands between 2004 and 2024. HBV = hepatitis B virus.

In the Netherlands, all acute cases diagnosed with a positive HBsAg test result and/or an anti-HBc IgM result are notifiable. Available samples of notified cases are requested and sent to the National Institute for Public Health and the Environment for sequencing. (Fig. 2) From 2004 to 2016, HBV in positive samples was sequenced based on the S and C gene, as described by Boot et al.^[8]^ From 2017 onward, sequencing is based on the near-complete genome of HBV.^[6]^ Between January 2004 and October 2024, a total of 2455 HBV-positive samples that were sequenced, including the strain from the case, were analyzed for mutations; 1879 had an acute infection, 474 had a chronic infection, and for 102, the infection status was reported as unknown. Genotype A2 is the most common genotype with 1288 (52.5%) cases, followed by genotype D with 507 (20.7%) cases during this period.

The D144A mutation has been identified in 4 other strains since 2004; 3 genotype A2 (2 chronic cases [2016 and 2018] and 1 with unknown infection status [2013]); and 1 genotype E (2006; chronic). Vaccination data were only available for 1 case (genotype E), who was unvaccinated. In 8 other cases, a D144E mutation was found (genotype D1 (n = 4, 2 acute, 1 unknown, 1 chronic) and genotype E (n = 4, all chronic). Vaccination data were available for 4 cases (genotype E), and all were unvaccinated. Other frequent HBsAg variants that include sites associated with vaccine escape in literature are P120T/S (n = 19), Q129HR (n = 15), and T/I126A/S/N (n = 14; Table 2). In total, 87 cases with an HBsAg variant were found (3.5%) with an average detection of 3.6% per year (range: 0% in 2008 to 6.5% in 2020). Individuals were predominantly male, with a male-to-female ratio of 3.2:1. The average age of cases with an HBsAg variant was 46 years. In total, vaccination data were available for 45 cases, only 2 of which were vaccinated, including a case with an HBsAg G145R variant (genotype F) and the case presented.

**Table 2:** HBsAg variants in the Dutch surveillance system with sites that have been associated with vaccine escape in literature.

Year	N sequences	Total HBsAg variants	% HBsAg variants	T116	P120	T126	Q129	M133	P142	D144	G145
2004	285	10	3.51%	**T116N**	**P120S** (2) **P120T** (2)	**T/I126A** **T/I126N**	**Q129R**			**D144E**	**G145A**
2005	326	14	4.29%	**T116N** T116R*	P120S (4*) P120T (2)	**T/I126A** T/I126N (2) **T/I126S**		**M133L** M133T		**D144E**	
2006	147	5	3.40%	T116P			**Q129N**	**M133L**		**D144A** **D144E**	
2007	103	1	0.97%				Q129P				
2008	126	0	0.00%								
2009	110	2	1.82%				**Q129R** (2)				
2010	108	4	3.70%			**T/I126A**	**Q129H** (2)	**M133L**			
2011	88	1	1.14%				**Q129H**				
2012	86	1	1.16%			**T/I126N**					
2013	116	5	4.31%				**Q129R**	M133T	P142L†	**D144A** † **D144E**	**G145R**
2014	108	5	4.63%		**P120T** (3)	**T/I126A**	**Q129R**				
2015	84	3	3.57%		P120T (2)		Q129P				
2016	107	4	3.74%	T116S			**Q129R**	M133Q		**D144A**	
2017	111	5	4.50%			**T/I126A**	**Q129R** ‡	M133T (2) M133I‡			**G145R**
2018	97	5	5.15%		**P120T** (2)		Q129H		P142L†	**D144A** † **D144E**	
2019	114	6	5.26%		**P120T** (2)	**T/I126S**	Q129H	**M133L** (2)			
2020	77	5	6.49%		**P120T**	**T/I126A** **T/I126S**		M133T		**D144E**	
2021	57	3	5.26%	**T116N**	**P120T** (2)						
2022	63	2	3.17%				**Q129R**	M133I			
2023	93	4	4.30%		**P120T**	**T/I126A** §	Q129P **Q129H**§		P142L§	**D144E**	
2024	49	2	4.08%							**D144A** **D144E**	
Total	2455	87	3.54%	6	18	14	18	13	3	13	3

HBsAg variants in the Dutch surveillance system that have in literature been associated with vaccine escape are bold, those with a substitution in the same location that have not been reported in literature are marked in normal typography.^[9]^

HBsAg = hepatitis B surface antigen.

* P120S/T116R was found as a double mutation in 1 sequence.

† P142L/D144A was found as a double mutation in 2 sequences.

‡ Q129R/M133I was found as a double mutation in 1 sequence.

§ T/I126A/Q129H/P142L was found as a triple mutation in 1 sequence.

## 3. Discussion

We present a patient with an acute HBV infection after full vaccination with a high post-vaccination anti-HBs control titer. Although our patient did develop acute hepatitis despite vaccination, he recovered quickly without treatment, indicating that the vaccination may have been protective against chronic infection. The most likely explanation for the symptomatic acute infection is the D144A mutation in the S gene of the patient’s HBV strain. This mutation has previously been described in other cases of vaccine escape.^[10,11]^ An alternative, theoretical explanation could be the waning of vaccine-induced immunity. However, a decline of anti-HBs to low or undetectable levels occurs frequently in vaccinated persons. In healthy persons, this is not regarded as a risk factor for symptomatic HBV infection, even after 30 years, as long as initial post-vaccination levels of anti-HBs were above 100 IU/L.^[12,13]^ Another explanation could be an already present chronic HBV infection before vaccination in 2003, with a current flare-up. However, the high signal for HBc IgM antibodies, the high anti-HBs titer following vaccination, the escape mutation in the S gene, and the quick spontaneous recovery make this unlikely.

Only a few clinically relevant mutations are known, which is partly explained by the good immune response of most hosts and overlapping reading frames in HBV.^[10]^ The HBV genome is highly compact, with about 50% of its genes overlapping in different reading frames. Therefore, a single nucleotide change can affect 2 or more viral proteins simultaneously, causing mutations to be potentially harmful to multiple essential viral functions. As a result, mutations in overlapping regions are less likely to persist, thereby reducing the emergence of clinically significant viral variants. HBsAg is the envelope protein that enables the virus to bind to hepatocytes. It contains the a-determinant, which is the most important epitope for neutralizing antibodies. This determinant has been mapped to amino acids 124-147 of the S-protein within the major hydrophobic region (99–170).^[14]^ This includes the D144A mutation found in our case. Antibodies induced by HBV vaccination are directed against this epitope. Mutations in this region can cause false negative results in HBsAg assays or allow evasion of anti-HBV immunotherapy or escape of vaccine-induced immunity.^[10,11]^ G145R is a well-known vaccine-induced immune escape mutation. Other mutations in the S-gene region include D144A, T116N, P120S/E, I/T126A/N/I/S, Q129H/R, M133L, and P142S.^[9]^

In our patient, HBV genotype A2 was found. This is an endemic strain in the Netherlands, and is identified in approximately 70% of acute and 35% of chronic cases in Dutch surveillance data.^[7,15]^ This genotype is most commonly found among MSM.

Mutations associated with immune or vaccine escape can affect vaccine efficacy and can pose a public health risk should they circulate more frequently. During the past 20 years of surveillance, no significant increase in vaccine escape mutations was seen.^[15]^ This is likely due to the fact that mutations rarely occur and generally negatively impact the virus’s survival. Furthermore, although HBV is highly contagious at the individual level, it is not very infectious at the population level, because there is a relatively low contact rate between infected and susceptible individuals as compared with, for example, respiratory viruses.^[16]^ Continuous monitoring, however, remains essential for identifying these cases and the public health context in which these mutations occur. Especially within the MSM community, where such strains theoretically may rapidly spread, vigilance remains important.

In conclusion, this case shows that after full vaccination, acute HBV infection may still occur due to vaccine escape mutations. Physicians should not rule out the possibility of an acute HBV infection in a vaccinated patient presenting with acute hepatitis. Surveillance is important to monitor the occurrence of such strains in the community, as they potentially pose a significant risk to public health.

## Acknowledgments

We would like to thank the technicians of IDS/RIVM, in particular Jeroen Cremer, for sequencing of the sample. The molecular surveillance of HBV is part of a broader HBV surveillance framework, including epidemiological surveillance. We would like to thank Tom Woudenberg, Anneke Westerhof, Francoise van Heiningen, and Irene Veldhuijzen for coordinating the epidemiological surveillance of HBV.

## Author contributions

**Conceptualization:** Sonja E. Leonhard, Roel P.J. Willems, Bettie C.G. Voordouw, Leonard C. Smeets.

**Data curation:** Sonja E. Leonhard.

**Investigation:** Daphne N. Fioole, Kimberley S.M. Benschop.

**Formal analysis:** Kimberley S.M. Benschop, Roel P.J. Willems.

**Supervision:** Mariska Petrignani, Leonard C. Smeets, Hans L. Zaaijer.

**Methodology:** Leonard C. Smeets, Kimberley S.M. Benschop.

**Writing – original draft:** Sonja E. Leonhard.

**Writing – review & editing:** Sonja E. Leonhard, Daphne N. Fioole, Kimberley S.M. Benschop, Roel P.J. Willems, Mariska Petrignani, Bettie C.G. Voordouw, Leonard C. Smeets, Hans L. Zaaijer.
